# Micro-Assembly Error Control of Specialized MEMS Friction Sensor

**DOI:** 10.3390/mi16020142

**Published:** 2025-01-26

**Authors:** Wei Zhou, Xiong Wang, Liwei Xue, Huihui Guo, Xiang Qin

**Affiliations:** 1 College of Information Engineering, Southwest University of Science and Technology, Mianyang 621010, China; zw222674@sina.com (W.Z.); xianqin26@gmail.com (X.Q.); 2 Hypervelocity Aerodynamics Institute, China Aerodynamics Research and Development Center, Mianyang 621000, China; 3 National Key Laboratory of Aerospace Physics in Fluids, Mianyang 621000, China; 4 School of Mechanical And Electrical Engineering, Soochow University, Suzhou 215000, China

**Keywords:** MEMS skin friction sensor, height error, line laser scanning, 3D reconstruction, hypersonic wind tunnel test

## Abstract

A skin friction sensor is a three-dimensional MEMS sensor specially designed for measuring the skin friction of hypersonic vehicle models. The accuracy of skin friction measurement under hypersonic laminar flow conditions is closely related to the fabrication and micro-assembly accuracy of MEMS skin friction sensors. In order to achieve accurate skin friction measurement, high-precision linear laser scanning ranging, multi-axis precision drive, and 3D reconstruction algorithms are investigated; a MEMS skin friction sensor micro-assembly height error measurement system is developed; and the MEMS skin friction sensor micro-assembly height error control method is carried out. The results show that the micro-assembly height error measurement of MEMS skin friction sensors achieves an accuracy of up to 2 μm. The height errors of the MEMS skin friction sensor were controlled within −8 μm to +10 μm after error control. The angular errors were controlled within the range of 0.05–0.25°, significantly improving micro-assembly accuracy in the height direction of the MEMS skin friction sensor. The results of hypersonic wind tunnel tests indicate that the deviation in the accuracy of the MEMS skin friction sensors after applying height error control is about 5%, and the deviation from the theoretical value is 8.51%, which indicates that height error control lays the foundation for improving the accuracy of skin friction measurement under hypersonic conditions.

## 1. Introduction

The skin friction of an aircraft’s surface is the main contributor to its total resistance, which can greatly limit the effective range and load of hypersonic vehicles. Studies show that the resistance of hypersonic vehicles in the atmosphere mainly includes skin friction resistance, differential pressure resistance, induced resistance, and surge resistance [1,2,3,4]. Moreover, the skin friction resistance experienced by hypersonic vehicles can account for up to 50% of their total resistance [5], directly limiting the effective range of these vehicles. In order to effectively suppress the skin friction of hypersonic vehicles, it is necessary to develop a high-accuracy skin friction measurement technology. High-fidelity CFD (Computational Fluid Dynamics) models of its aerodynamic shape can be established in the design phase, and wind tunnel tests of the prototype can be conducted in the development phase to achieve the design verification and validation of the CFD model and the prototype.

Skin friction measurement methods are divided into indirect methods [6,7] and direct methods [8,9]. Indirect skin friction measurement is mainly performed through the acquisition of data concerning heat exchange, velocity gradient, pressure gradient, and other parameters; substituting these data into the measurement model for calculation; and indirectly obtaining the value of skin friction. Direct measurements in hypersonic wind tunnels mainly depend on various conventional skin friction balances [10,11,12,13]. Typically, direct measurements use the principle of force equilibrium, where a floating element is positioned directly on the surface, and the amount of deformation of the floating element is measured; the amount of deformation can then be calibrated in advance in relation to the force to obtain an accurate skin friction value [14,15].

The MEMS skin friction sensor we developed is designed based on the direct measurement method and MEMS technology. In our design, the floating element is flush with the wall to be measured, and the signal output microstructure is isolated from the flow field of a hypersonic wind tunnel. Validation tests indicated that the sensor has an accuracy deviation in the range of 11.75–23.60% [16], a stable signal measured by the sensor during the wind tunnel test, zero drift, high sensitivity, and repeatability better than 2%, providing a viable experimental research tool for skin friction measurements in hypersonic flow fields. The sensor is a three-dimensional MEMS sensor with high assembly requirements, for which a dedicated vision-based micro-assembly system has been developed. The concentricity error of the assembled sensor’s floating element and the circular hole of the package cover is up to 2 μm [17]. In hypersonic laminar flow conditions, micro-assembly errors in the height direction can cause the measured skin friction of the sensor to deviate from the theoretical value by up to 100% [18]. To ensure that the measured value of the MEMS skin friction sensor deviates from the theoretical value by no more than 10%, the micro-assembly height error of the MEMS skin friction sensor should be controlled within the range of −20 μm to 10 μm [19]. In order to achieve accurate skin friction measurement, it is urgent to carry out research on the floating element and package cover assembly of MEMS skin friction sensors as well as on the accurate measurement and control of the height errors of the sensor’s installation on the wall to be measured.

## 2. Analysis of Measurement and Control Methods

### 2.1. Analysis of Sensor and Its Micro-Assembly Height Error Characteristics

**Figure 1:**
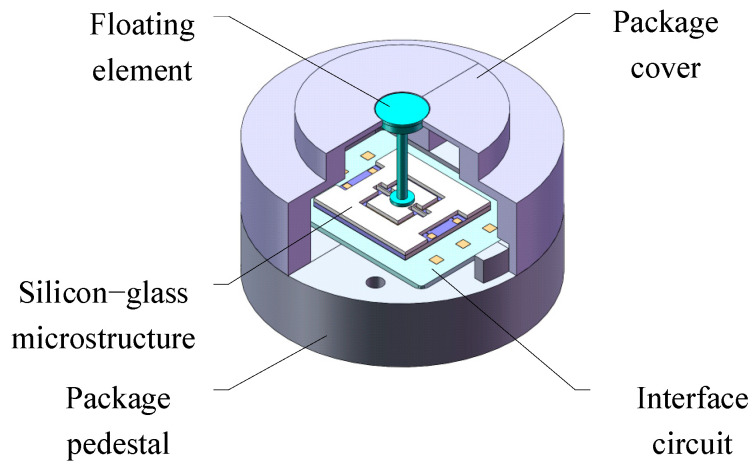
Composition diagram of MEMS friction sensor.

The MEMS skin friction sensor of the aforementioned design adopts a mesoscopic three-dimensional sensitive structure with a floating element flush with the wall to be measured and the signal output microstructure isolated from the flow field of the hypersonic wind tunnel. As shown in Figure 1, it mainly consists of a floating element, silicon–glass microstructure, ceramic-based interface circuit, package cover, and package pedestal.

The silicon–glass microstructure is a MEMS microstructure in which a single crystal of silicon is etched and anodically bonded to the glass electrode; the floating element, package cover, and package pedestal are made via a precise processing process; the silicon–glass microstructure is bonded to the metal-plated side of ceramic-based interface circuit by dispensing; the ceramic-based interface circuits, silicon–glass microstructures and floating elements are pre-assembled; positional tolerance of the micro-assembly is ensured by means of locating surface and hole on the shaft, which is fixed by dispensing. High-accuracy micro-assembly of MEMS skin friction sensor in the horizontal direction is achieved by using the vision alignment-based micro-assembly system that identifies and aligns the center of the circle of the floating element and the circular hole of the package cover, and then the silicon–glass microstructure is bonded to the metal-plated side of ceramic-based interface circuit by dispensing it to the assembly position of the package pedestal.

**Figure 2:**
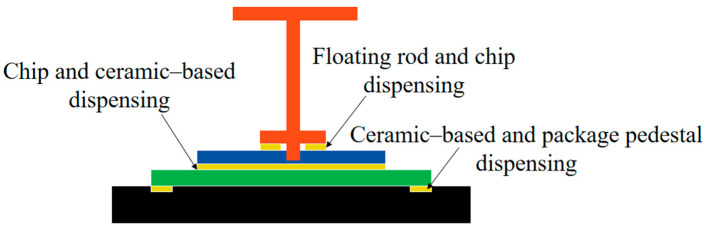
Schematic diagram of dispensing position of MEMS friction sensor.

The MEMS skin friction sensor is mainly assembled in a series of floating elements, silicon–glass microstructure, ceramic-based interface circuit, package pedestal, and package cover. Processing errors in the height direction exist between every component, and the silicon–glass microstructures are bonded to the metal-plated side of the ceramic-based interface circuit by dispensing together, as shown in Figure 2.

**Figure 3:**
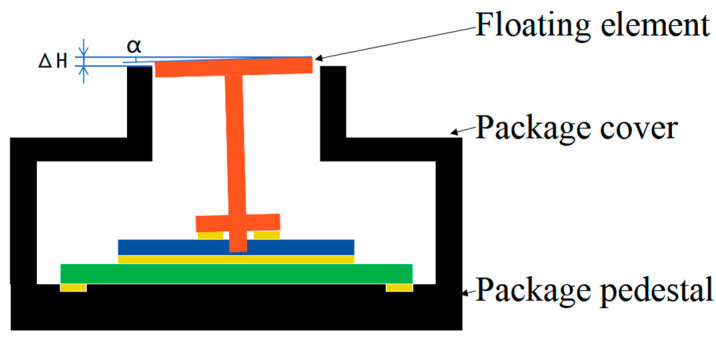
Schematic diagram of height and angular errors between the floating element and encapsulation cover plate.

The uncertainty of the thickness of the multi-coated adhesive layer leads to a certain height error between the floating element of the MEMS skin friction sensor and the upper surface of the package cover after assembly (a bulge is positive tolerance, and concave is negative tolerance), and the accumulated height error can be up to 100 μm. At the same time, because there are certain flatness errors in the processing of each component and the unevenness in the thickness of the multi-coated adhesive layer, it results in certain angular errors between the floating element and the upper surface of the package cover, as shown in Figure 3. During the wind tunnel test, the MEMS skin friction sensor is installed flush with the wall to be measured by the test model due to the existence of processing errors, and the measuring surface of the MEMS skin friction sensor has a certain height error with the wall to be measured. These accumulated assembly errors have a significant impact on the accuracy of skin friction measurements and need to be accurately measured and controlled.

### 2.2. Accurate Measurement and Control Methods

The aforementioned analysis indicates that in order to achieve accurate skin friction measurement (the deviation between the measured value and the theoretical value of the MEMS skin friction sensor is less than 10%), MEMS skin friction sensor micro-assembly height errors should be controlled between −20 μm and 10 μm [8], and the measurement accuracy should be better than 5 μm. Since the size of the torsion beam of the MEMS skin friction sensor is only in the order of a hundred μm, the floating element of the MEMS skin friction sensor needs to avoid being in contact during the measurement process; otherwise, the sensitive structure of the MEMS skin friction sensor will be easily damaged. Therefore, MEMS skin friction sensor micro-assembly height error measurement is realized using non-contact line laser precision scanning (with a height direction measurement accuracy better than 1 μm) and a high-accuracy 3D reconstruction algorithm.

**Figure 4:**
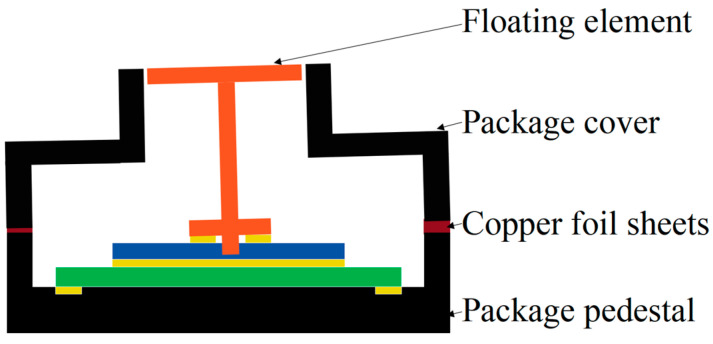
Schematic diagram of height and angular errors revised between the floating element and encapsulation cover plate.

The floating element, silicone–glass microstructure, ceramic-based interface circuit, and package pedestal are all bonded by dispensing. Height errors exist between the floating element and the upper surface of the package cover, and the distance control between the package cover and the package pedestal in the height direction can only be achieved by the design of the floating element slightly protruding out of the package cover. The height error between the floating element and the upper surface of the package cover is controlled by placing copper foil sheets with thicknesses of 50 μm, 20 μm, and 10 μm at symmetrical positions between the package cover and the package pedestal. The angular error of the floating element is controlled against the upper surface of the package cover by padding copper foil sheets and controlling the installation error of the MEMS skin friction sensor in the height direction with copper foil sheets between the installation step of the package cover and the installation surface of the wall to be measured in the test model, as shown in Figure 4.

## 3. Research on the Accurate Measurement Technique

Analysis reveals that the key techniques of MEMS skin friction sensor micro-assembly height errors accurate measurements include the following: (1) High-precision line laser scanning—line laser scanning is based on laser triangulation, where the relative positions of the laser measuring instrument and the point laser rangefinder are calibrated, and the scanning height between the line laser scanner and the object to be measured can be dynamically adjusted by the measured value of the point laser. (2) Precision positioning platform—positioning technology in the micron range is achieved by means of a servo-positioning platform or other drives; precise positioning is achieved by building a linear motor module platform and compensating for it with a higher-precision laser interferometer, ensuring the precise splicing of the line laser scanning profile. (3) A 3D reconstruction algorithm—fast and highly accurate acquisition of 3D information in the height direction of the measurement object is achieved according to the defined scanning range. (4) Adaptive cloud map display—splicing multi-view point clouds by using the positioning accuracy of the precision mobile platform itself and compensating for rigid splicing errors. The MEMS skin friction sensor micro-assembly height error measurement system was designed using the above key technologies.

**Figure 5:**
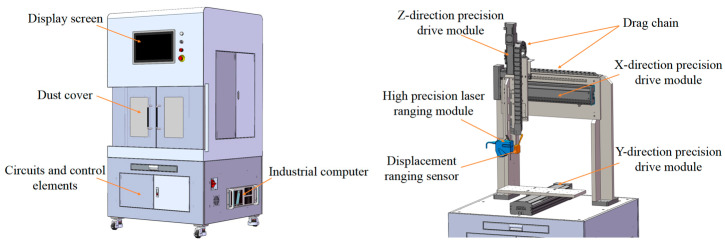
MEMS friction sensor micro-assembly and installation height error measurement system.

Figure 5 shows the composition of the MEMS skin friction sensor micro-assembly height error measurement system, which contains a precision positioning fixture, a precision positioning platform, a line laser scanning system, a 3D reconstruction, and an adaptive cloud map display system. The precision positioning fixture ensures that MEMS skin friction sensors stay in place during measurements. The precision positioning fixture ensures that MEMS skin friction sensors stay in place during measurements. The precision positioning table is used for the precise handling of sensors during inspection. The 3D reconstruction algorithm is used to quickly and accurately acquire 3D information about the surface topography of an object based on a defined scanning range. The cloud map display system, based on the acquisition of high-precision 3D data, extracts the characteristics of the required measurements for the display of 3D information within the scanning range and facilitates access to the local area of the height error information. The system is summarized as consisting of three main tasks: (1) form and position assurance during device measurement; (2) the precise identification and localization of feature positions via line laser scanning; (3) the realization of 3D reconstruction and cloud map display.

During the measurement process, the sensor is placed horizontally on the precision positioning platform, and the alignment and scanning of the sensor by the line laser scanning system are achieved through X/Y/*Z*-axis movement, the 3D reconstruction of the sensor surface topography is completed after the scanning, and then the result is presented on the display screen through the adaptive cloud map display system.

### 3.1. High-Precision Line Laser Scanning Measurement Method

As the sensitive structure of MEMS skin friction sensor adopts the brittle material of single crystal silicon micro-beam chip structure, the contact measurement method is very likely to lead to the fracture damage of the sensor’s elastic beam, while the non-contact measurement method does not cause any damage. The non-contact measurement method has a high data acquisition rate, which makes it easy to acquire a large amount of data without the need for point-by-point measurement. It also has a high efficiency of coordinate acquisition.

**Figure 6:**
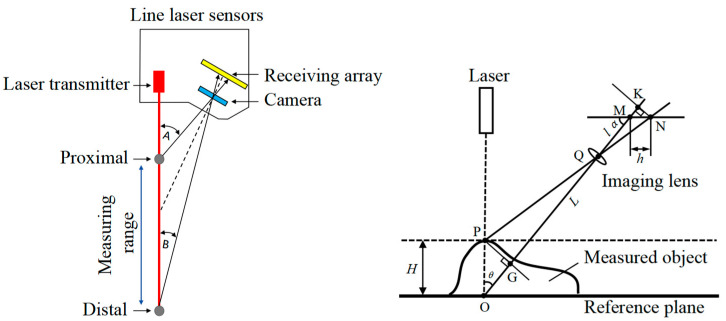
The principle of distance measurement using laser triangulation.

Line laser scanner acquires the advantage of high measurement accuracy and high scanning frequency, and the ranging principle is laser triangulation, as shown in Figure 6. Laser triangulation is an active measurement method. By fixing the relative positions of the camera and the laser transmitter, we can collect the light information emitted by the laser transmitter; the changes in the shape of the surface of the object will be reflected in the received picture of the light spot, causing its position to be shifted, and the size of this shift corresponds to the change in the depth of the surface of the object, so the offset pixel value of the light point on the picture can be calculated, and thus the depth information of the object surface can be obtained indirectly. The light transmission route is triangular in space, so it is called laser triangulation. A point laser rangefinder is set near the line laser profile measuring instrument. Before laser scanning, the displacement rangefinder sensors first scan and obtain the workpiece surface profile, obtain the distance data between each point of the profile and the laser receiving surface, obtain the *Z*-axis position during scanning through calculation, and adjust the height of the laser measuring instrument via the control program to ensure that each point of the workpiece’s surface is within the scanning range of the laser measuring instrument. After calibrating the relative positions of the line laser profile measuring instrument and the point laser rangefinder, the scanning height between the line laser scanner and the measured object can be dynamically adjusted using the measured value of the point laser, which can be adapted to scanning objects of different heights. When measuring the height error of the sensor, the sensor is first placed horizontally on the platform, and the floating element has a height error relative to the upper surface of the package cover. The sensor surface is scanned by the laser scanner to obtain the height data of different scanning points on the sensor’s surface, and the height error map of the entire sensor surface is obtained through subsequent processing.

**Figure 7:**
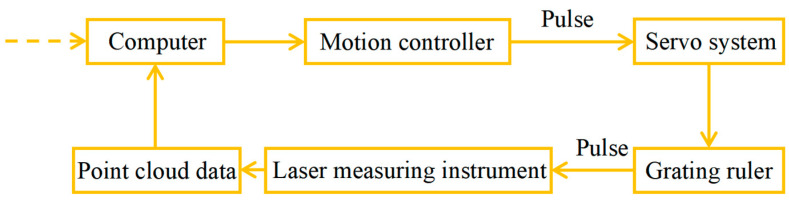
Schematic diagram of height error measurement of MEMS skin friction sensor.

A certain type of laser profile measuring instrument with a resolution of 2.5 μm in the X and Y directions and a repeatability of 0.3 μm in the depth direction is chosen in this study. The accuracy of this instrument can serve as an indicator for measuring the height error of the MEMS skin friction sensor. In terms of the task of measuring the sensor assembly height errors using the laser measuring instrument and reconstructing the 3D texture features of the sensor’s rough surface, the measurement process is shown in Figure 7.

The motion controller, with the help of the computer, sends pulse commands to control the movement of the servo system. When there is a micron-level relative displacement between the measured object and the laser measuring instrument, and the motion controller generates a certain number of square, rectangular waves and sends pulse signals to trigger the laser measuring instrument to collect the scanning point cloud data, the computer uses the three-dimensional reconstruction algorithm to obtain the skin friction sensor assembly height errors and surface roughness and topography information. In addition to precise position positioning via the precision platform of the linear module, the rough topography measurement system of the MEMS skin friction test also needs to fix the position of the sensor during measurement. Since part of the topography data can be used to determine the relative position between the measurement planes, the repeated positioning accuracy of the sensor in the device is not too high, so the side wall of the MEMS friction sensor base is used for positioning.

### 3.2. Precision Positioning Platform for Height Error Measurement

**Figure 8:**
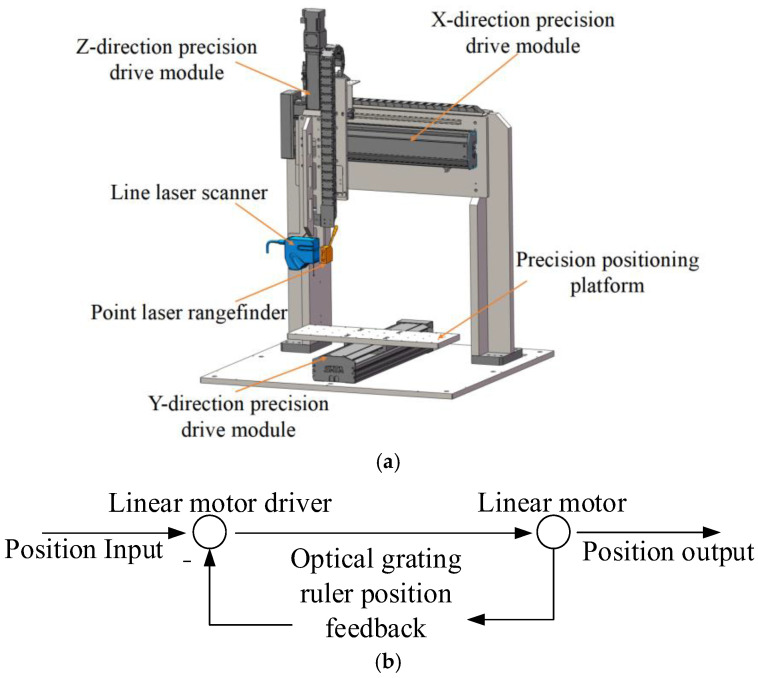
Precision positioning platform. (**a**) Platform structure; (**b**) control principle.

The structure diagram and control principle of the precision positioning platform are shown in Figure 8. To achieve the precision handling and assembly of various components, the X/Y/Z three-axis precision positioning platform is built using a linear motor + slider rail + grating ruler. After laser interferometer calibration, the position information is fed back through the grating ruler reading head, the positioning accuracy of the precision positioning platform is less than 5 μm, and the repeated positioning accuracy is 2 μm. When the grating ruler is installed on the *X*-axis, and there is a relative displacement of microns between the measured object and the laser measuring instrument, a certain number of square, rectangular waves are generated, and pulse signals are sent to trigger the laser measuring instrument to collect the scanning point cloud data.

### 3.3. 3D Reconstruction Algorithm

**Figure 9:**
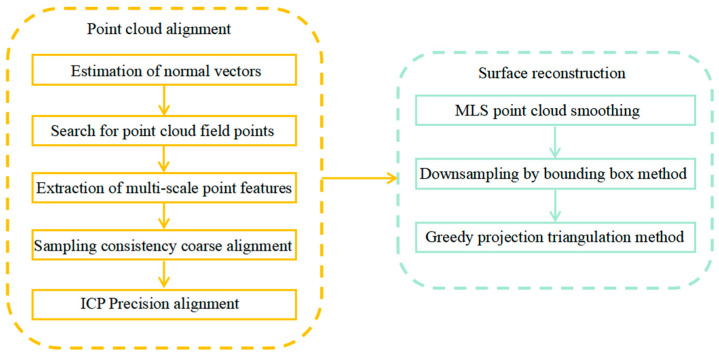
Flow chart of 3D reconstruction algorithm.

Measuring the height errors in the micro-assembly of MEMS skin friction sensors requires the reconstruction of 3D information of the scanned area. The hardware equipment needs to be calibrated before laser scanning: (1) calibration of internal and external camera parameters; (2) calibration of laser plane; (3) motion calibration of moving slides. After the calibration, the position coordinates of the workpiece are obtained by laser scanning, the depth information is obtained by software calculation, and the surface of the model is reconstructed in three dimensions to obtain the texture features of the surface roughness. The 3D reconstruction algorithm process mainly consists of point cloud alignment and surface reconstruction, as shown in Figure 9.

#### 3.3.1. Point Cloud Acquisition and Denoising

Due to the limitations of the laser equipment and the environment, it may not be possible to obtain the complete data at one time when sampling the data points of the workpiece and obtain the partial point cloud data of the workpiece in batches. MEMS skin friction sensor micro-assembly height error measurement requires a large measurement range and high measurement accuracy. Considering the existing measurement methods comprehensively, a contradiction between the measurement range and the measurement accuracy also exists. Therefore, a line laser scanner is used to obtain high-precision multi-view point cloud information, and then the multi-viewpoint cloud information is spliced together to obtain high-precision information on a wide range of surface topography. Noise in point cloud data is unavoidable, and the main point cloud noise obtained by line laser scanner includes the following: (1) drifting points—sparse, scattered points that float above the point cloud, obviously far from the main body of the point cloud; (2) isolated points—small, dense point clouds far from the center of the point cloud; (3) redundant points—extra scan points beyond the intended scan area; (4) mixed points—the noise points that are mixed up with the correct point cloud. Therefore, the point cloud data need to be filtered and denoised before splicing.

**Figure 10:**
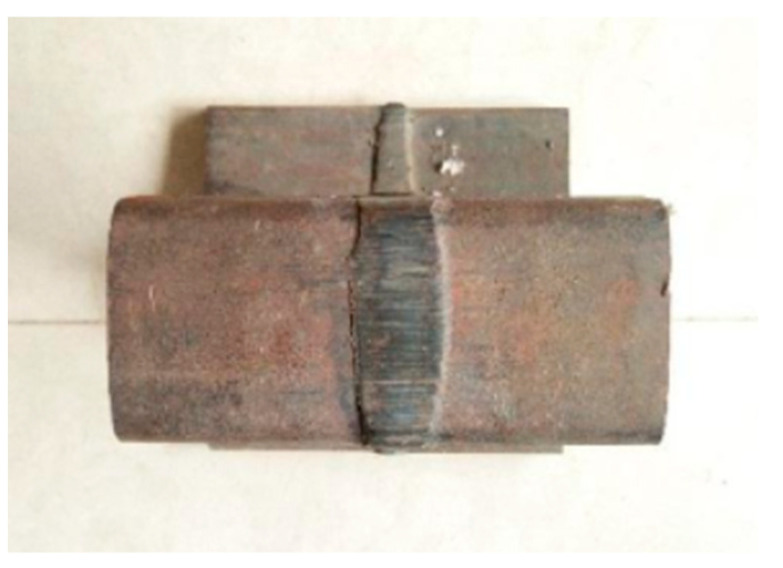
The photo of welding seam.

**Figure 11:**
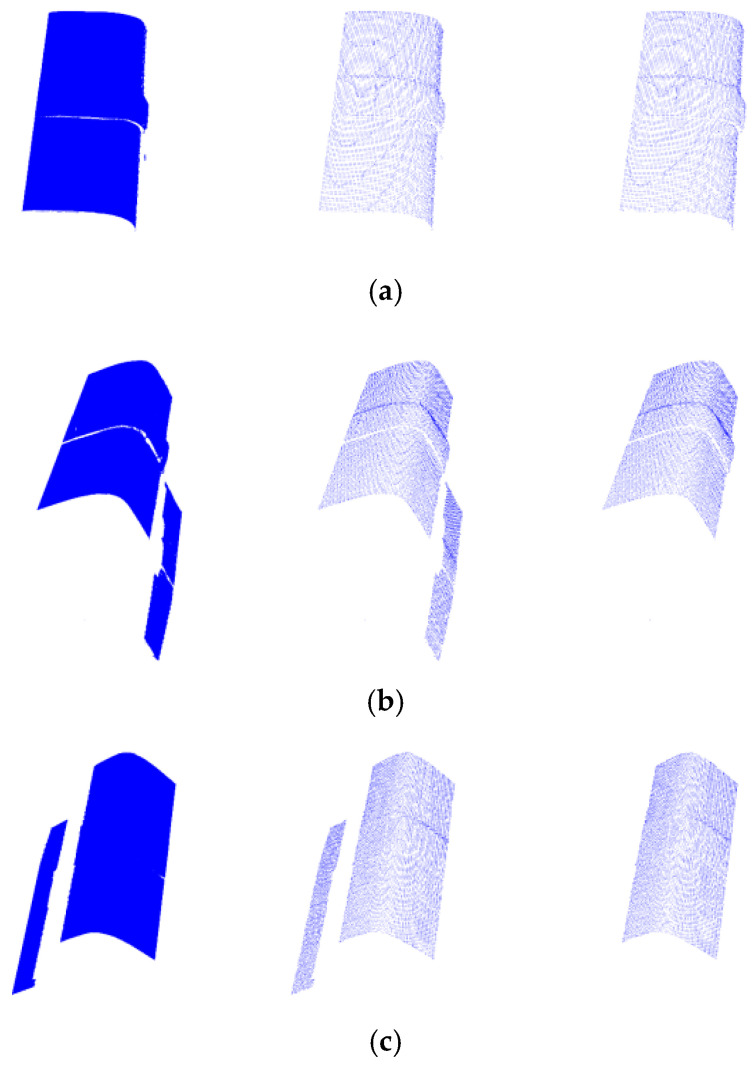
Point cloud data of the welding seam from different perspectives. (**a**) Point cloud data from perspective 1; (**b**) point cloud data from perspective 2; (**c**) point cloud data from perspective 3.

Take the welding seam (Figure 10) as an example for multi-view data acquisition; obtain the point cloud data from perspectives 1, 2, and 3 as shown in Figure 11; and denoise the point cloud.

#### 3.3.2. Point Cloud Alignment and Fusion Splicing

**Figure 12:**
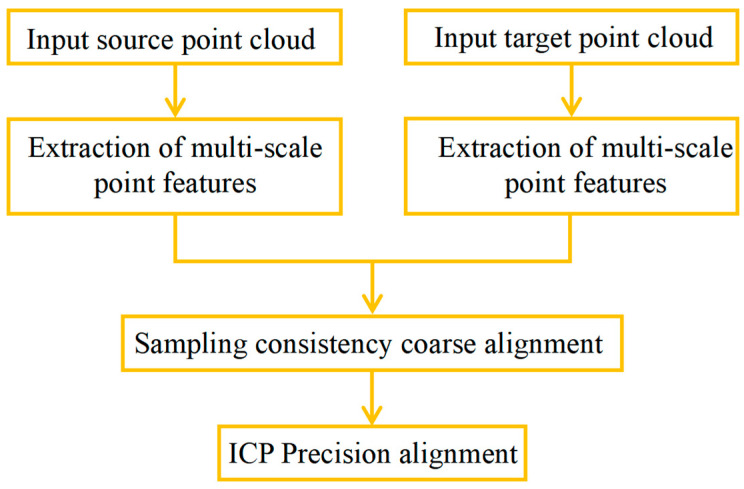
Point cloud registration method based on multi-scale point features.

When acquiring point cloud data in batches, the coordinate system in which different batches of data are located changes, and there are translation misalignments and rotation misalignments. In order to unify all the data under the same coordinate system, it is necessary to find the correspondence of the points between different point clouds and then compute the translation and rotation matrices so as to map one set of point clouds to another set of point clouds. This process is point cloud alignment. By adopting the point cloud alignment method based on multi-scale point features, including estimating normal vectors, searching point cloud domain points, extracting multi-scale point features, adopting consistency coarse alignment, and adopting ICP (iterative closest point) fine alignment, we can obtain a model with high alignment accuracy, and, at the same time, achieve high alignment efficiency, as shown in Figure 12.

Point cloud alignment and fusion are used to splice the multi-viewpoint cloud generally, and the features of the point cloud data, such as feature points and normal vectors, are used to establish the position transformation relationship between the point clouds. Since the surface of the scanned object is nearly flat, there are very few features that can be used for alignment, so the alignment result is unstable. Thus, the multi-viewpoint cloud is spliced by utilizing the positioning accuracy of the precision mobile platform itself as well as compensating for the rigid splicing error.

**Figure 13:**
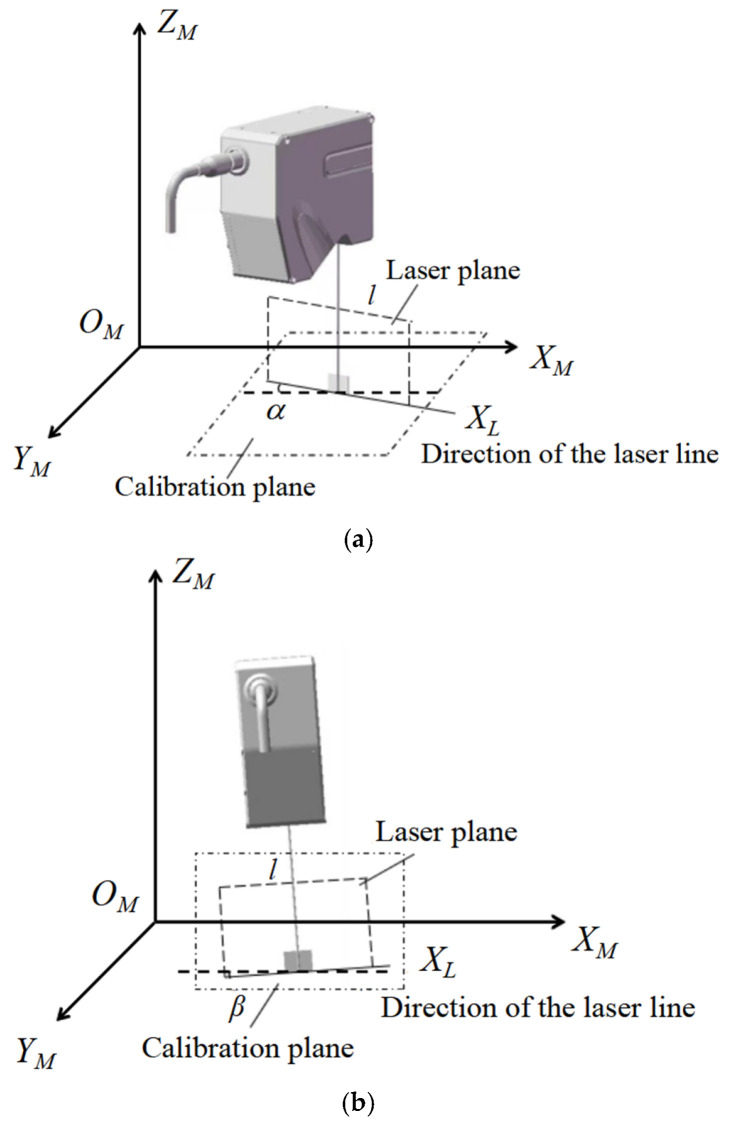
Line laser scanner installation errors in rigid splicing error analysis. (**a**) Installation error between the laser plane and calibration plane of line laser scanner; (**b**) installation error between the height baseline and calibration plane of line laser scanner.

On the basis of scanning with a precision mobile platform, splicing errors are mainly caused by installation errors of the line laser scanner. Two of the main installation errors include (as shown in Figure 13) the following: (1) the intersection line of the line laser profile scanner’s laser plane and calibration plane OMXMYM is not parallel to the direction of the OMXM axis in the reference plane; (2) the height baseline of the line laser profile scanner is not parallel to the direction of the OMXM axis in the calibration plane OMXMYM. When the point cloud is spliced together, accurate and complete surface topography information is obtained by compensating for these two installation errors. On the basis of the complete surface topography information, the roughness of the surface within the scanning range can be calculated, and the highest and lowest positions of the extracted area can be measured.

**Figure 14:**
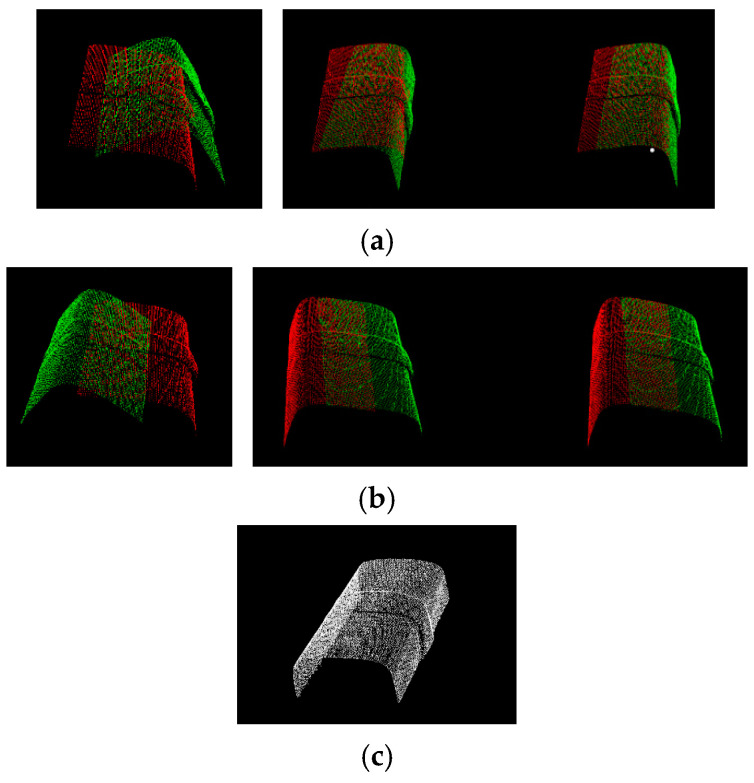
The point cloud data of welding seam from perspectives 1, 2, and 3. (**a**) Initial positions and registration of point cloud data from perspective 1 and perspective 2; (**b**) initial positions and registration of point cloud data from perspective 1 and perspective 3; (**c**) the result of point cloud data registration from perspective 1.

The welding seam will utilize the point cloud data of three perspectives after downsampling and filtering processing for the alignment experiments. The two-by-two alignment method is adopted, the point clouds of perspectives 1 and 2 as shown in Figure 14a and perspectives 1 and 3 as shown in Figure 14b are, respectively, aligned two-by-two. Finally, the alignment results will be uniformly transformed to the coordinate system of perspective 1 as shown in Figure 14c.

#### 3.3.3. Surface Reconstruction Method

**Figure 15:**
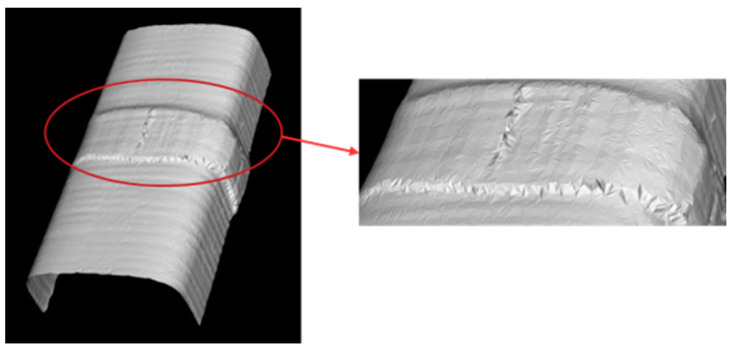
The 3D reconstruction results from perspective 1 coordinate system.

After preprocessing and alignment, the point cloud data obtained by the laser rangefinder are still discrete data points on the surface of the object, which lacks the topological relationship between the data points and requires surface reconstruction to obtain the surface morphology of the object. The normal vector is first smoothed by using the MLS (Moving Least Squares) method; then, downsampling of the data is performed by using the bounding box method to reduce calculation cost and increase reconstruction speed; finally, the greedy projection triangulation method is used to filter the points by multi-neighborhood criterion to form a triangular mesh mapped back to the 3D space to complete the 3D reconstruction process. The point cloud data of perspective 2 and perspective 3 are aligned and unified to the coordinate system of perspective 1 (Figure 15), and the model of the welding seam can be obtained using the bounding box algorithm to streamline it. The aligned and streamlined point cloud model is surface-reconstructed using the greedy projection triangulation method, and the 3D model is obtained.

#### 3.3.4. Calculation of Height Errors and Angular Errors

**Figure 16:**
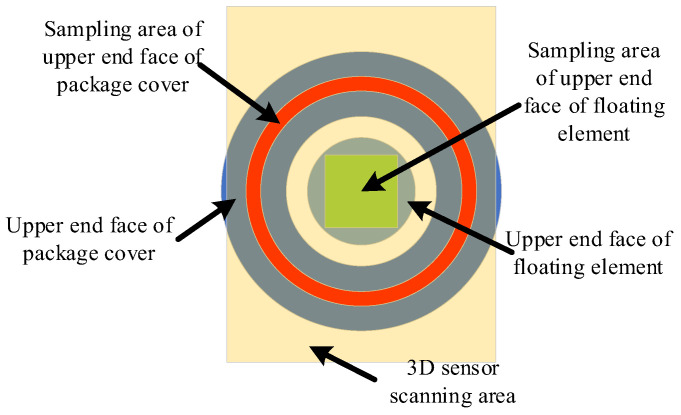
Schematic diagram of MEMS skin friction sensor scanning and sampling area.

The reconstructed 3D point cloud model of the sensor has a high recovery accuracy, and the accuracy of the attitude information between the upper end face of the floating element and the upper end face of the package cover is high. In this study, 1000 sets of uniform samples are used in the square area of the floating element of the sensor, and 3600 sets of samples are used in the ring belt of the upper package cover. Figure 16 shows the schematic diagram of sampling.

The plane fitting algorithm was applied to the above two sets of data samples. The least squares algorithm was utilized to obtain the two plane equations, respectively. The relative minimum spatial angle between the two planes was calculated indirectly to obtain the angular error. The floating element area is divided into equal angle sections for point sampling, and the relative distance with respect to the upper package cover end face is calculated separately to characterize the height error of the floating element end face with respect to the upper package cover end face.

The method of the point fitting plane is to square the difference between the fitted value of the point and the plane to find its minimum value. The parameters of the corresponding fitted plane are the coefficients of the plane expression. The minimum value is obtained from Formula (1).

(1) $$S=\overset{n}{\underset{i=0}{\sum }}{\left(a_{0}x_{i}+a_{1}y_{i}+a_{2}-z_{i}\right)}^{2},$$

$a_{i}\left(i=0,1,2\right)$ is the plane equation parameter, which is used as a variable here. A partial derivative is applied to it to calculate $\frac{\partial S}{\partial a_{i}}$, obtaining Formula (2).

(2) $$\left\{\begin{array}{c} \sum 2\left(a_{0}x_{i}+a_{1}y_{i}+a_{2}-z_{i}\right)x_{i}=0\\ \sum 2\left(a_{0}x_{i}+a_{1}y_{i}+a_{2}-z_{i}\right)y_{i}=0\\ \sum 2\left(a_{0}x_{i}+a_{1}y_{i}+a_{2}-z_{i}\right)=0 \end{array}\right.,$$

The formula can be further rewritten as a matrix Formula (3).

(3) $$\left[\begin{array}{ccc} \sum {x_{i}}^{2} & \sum x_{i}y_{i} & \sum x_{i}\\ \sum x_{i}y_{i} & \sum {y_{i}}^{2} & \sum y_{i}\\ \sum x_{i} & \sum y_{i} & n \end{array}\right][\begin{array}{c} a_{0}\\ a_{1}\\ a_{2} \end{array}]=\left[\begin{array}{c} \sum x_{i}y_{i}\\ \sum y_{i}z_{i}\\ \sum z_{i} \end{array}\right],$$

The plane formula parameter $a_{i}\left(i=0,1,2\right)$ is obtained.

**Figure 17:**
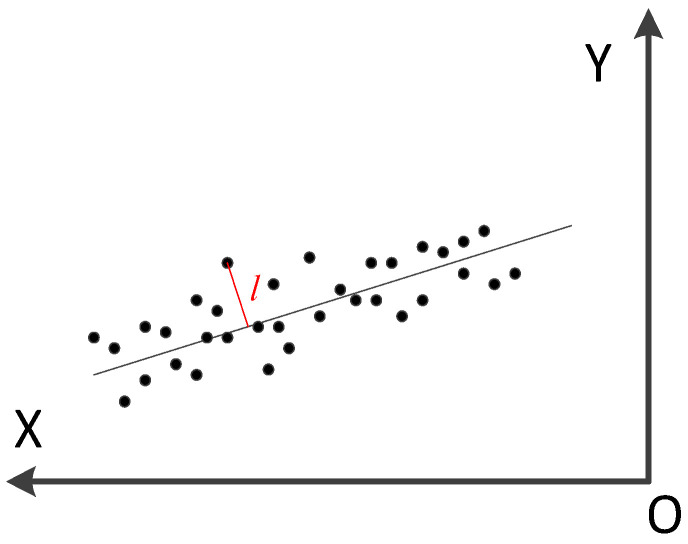
Sectional schematic diagram of space point exclusion.

In order to obtain more accurate plane characterization equation parameters, it is necessary to filter out the fitted points once before the plane-fitting parameters are determined. The specific method is to pre-fit the plane to the data points involved in the calculation, calculate the distance for each sampling point, and eliminate the points exceeding the expected value before performing the final plane fit to the remaining points. The schematic diagram of space point culling interface is shown in Figure 17.

## 4. Height Error Measurement and Control of MEMS Skin Friction Sensors

### 4.1. Measurement of Height Errors and Angular Errors

**Figure 18:**
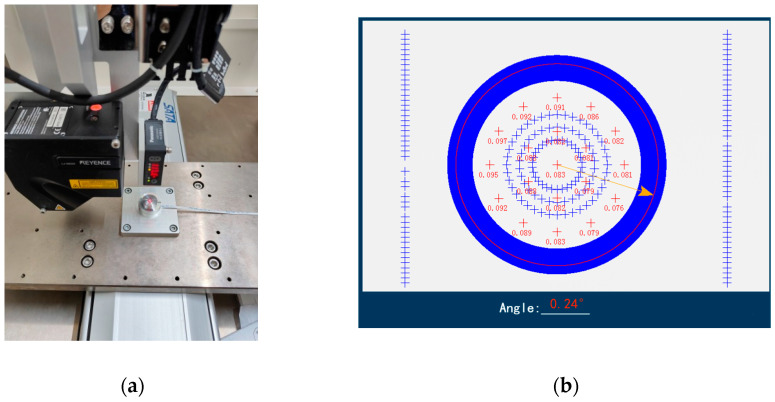
Height error measurement. (**a**) The photo of sensor height error measurement; (**b**) the result of #3 sensor height error measurement.

The physical photographs of the height error measurement after sensor micro-assembly are shown in Figure 18. Based on these figures, the height errors and angular errors of the sensor assembly were measured, respectively. The measurement results of a set of MEMS skin friction sensors are shown in Table 1, where the positive value indicates that the upper end face of the floating element is above the upper end face of package cover, and the negative value indicates that the upper end face of floating element is below the upper end face of package cover. It can be seen that due to the influence of processing errors and the accumulation of errors in the micro-assembly of multiple gluing and pasting, the height errors between the measurement surface of the floating element and the package cover are in the range of 26 μm–123 μm, and the angular errors are in the range of 0.15–0.47°, which are not in line with the aforementioned requirements for surface friction measurement under the conditions of hypersonic speed.

### 4.2. Height Errors Control

**Figure 19:**
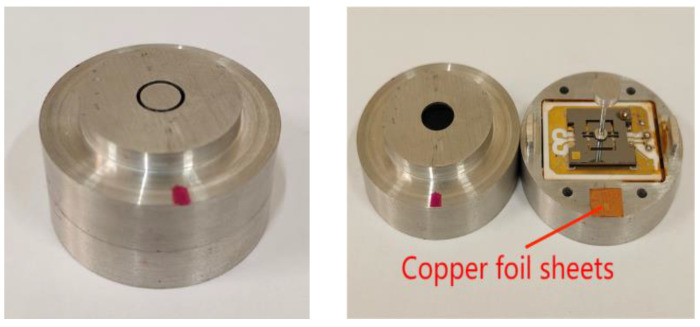
The #3 sensor height error control.

To cope with the effect of sensor micro-assembly error on the sensor height difference, the height error control of the sensor is performed, as shown in Figure 19. The method of adding copper foil sheets between the package cover and the package pedestal is adopted. According to the measurement results in Table 1, where the upper end face of the floating element is higher than the upper end face of the package cover is marked, and then the copper foil sheets are added to the mark successively to revise the height error. The height error control results are shown in Table 2.

**Figure 20:**
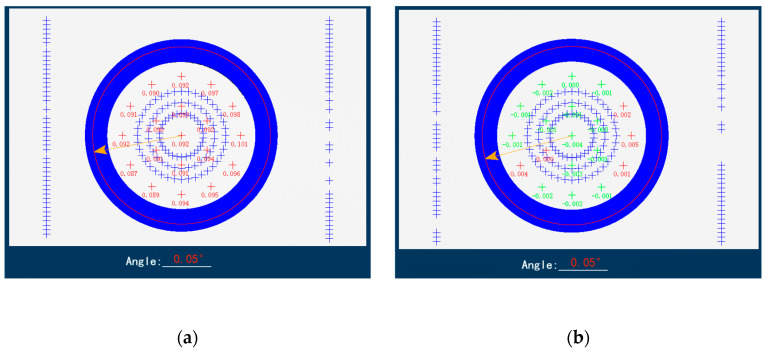
The height error comparison of #3 sensor. (**a**) The measurement results of #3 sensor before control; (**b**) the measurement results of #3 sensor after control.

The results indicate that the height error range of the sensor after error control is −8 μm–10 μm, and the angular error range is 0.05–0.25°, which meets the requirement that the height difference between the upper end face of floating element and the upper end face of the package cover is ±10 μm. The comparison of results measured before and after controlling the height error of the 3# sensor is shown in Figure 20.

## 5. Validation in a Hypersonic Wind Tunnel

Validation of the sensor prototype in a hypersonic wind tunnel mainly involves prototype screening and repeatability tests to obtain the accuracy of MEMS skin friction sensors. Sensor prototype and test facility are fixed in the working section of the Φ1 m hypersonic wind tunnel of CARDC. The Mach number is M_∞_ = 8, the angle of attack for the model is α = 0°, and the detailed flow field conditions are presented in Table 3.

The verification tests under laminar flow are implemented at P_0_ = 2.5 MPa conditions, and the sensor skin friction coefficient obtained from the test is shown in Table 4.

The result indicates that the accuracy deviation of the MEMS skin friction sensors after height error control is about 5%, and the deviation from the theoretical value is 8.51%. Compared with the previous batch of sensors, the repeatability and accuracy of test data have been improved.

## 6. Conclusions

The analysis of the MEMS skin friction sensor micro-assembly’s height error and measurement requirements is presented in this paper. The high-precision line laser scanning measurement system is researched. A precision positioning platform for the height error measurement of the MEMS skin friction sensor is designed. The 3D reconstruction algorithms, including the point cloud acquisition, the point cloud alignment and fusion splicing, the surface reconstruction, and the acquisition of the height errors and angular errors are researched. The micro-assembly height error measurement equipment for the MEMS skin friction sensor is developed, and the equipment is utilized to measure the micro-assembly height errors and angular errors of the MEMS skin friction sensor, on the basis of which the height errors and angular errors of the floating element existing on the upper end face of the package cover and the package pedestal are controlled by means of padding the copper foil sheets at the symmetric position between the package cover and the package pedestal.

The results indicate that the height direction error measurement accuracy of micro-assembly height error measurement equipment is better than 2 μm. The height errors between the floating element and the package cover are controlled in the range of −8 μm to +10 μm by using copper foil sheets. The angular errors are within the range of 0.05–0.25°, and the micro-assembly accuracy of MEMS friction sensor is improved. The result of hypersonic wind tunnel tests indicates that the deviation in the accuracy of the MEMS skin friction sensors after applying height error control is about 5%, and the deviation from the theoretical value is 8.51%, which shows that height error control lays the foundation for improving the accuracy of skin friction measurement under hypersonic conditions.

## Figures and Tables

**Table 1:** Height error measurement results.

Sensor Number	Low-Point Error (μm)	High-Point Error (μm)	Angular Error (°)
1	26	59	0.47
2	26	40	0.20
3	76	97	0.24
4	87	101	0.15
5	82	115	0.47
6	103	123	0.25

**Table 2:** Height error control results.

Sensor Number	Low-Point Error (μm)	High-Point Error (μm)	Angular Error (°)
1	−5	7	0.15
2	−8	3	0.17
3	−3	5	0.10
4	−2	5	0.05
5	−5	10	0.25
6	−7	5	0.24

**Table 3:** The detailed flow field parameters of the wind tunnel used for sensor validation.

Nominal Mach Number	Total Pressure P_0_ (Pa)	Total Temperature T_0_ (K)	Static Pressure P_∞_ (Pa)	Dynamic Pressure q_∞_ (Pa)	Unit Reynolds Number Rel (m^−1^)
8	2,526,169.486	711.102	256.663	11,527.256	5.95 × 10^6^
	2,515,718.296	725.438	255.601	11,479.566	5.72 × 10^6^
	2,502,958.206	726.351	254.304	11,421.340	5.68 × 10^6^
	2,492,612.280	720.873	253.253	11,374.130	5.73 × 10^6^
	2,496,346.624	724.421	253.633	11,391.170	5.69 × 10^6^

**Table 4:** Hypersonic wind tunnel test data.

Angle of Attack (°)	Skin Friction Coefficient Measured by 7 MEMS Skin Friction Sensors							Average Value	Accuracy Deviation	Brasius Theoretical Solution	Theoretical Deviation
	2#	4#	6#	7#	8#	11#	12#				
0	0.00066	0.00063	0.00067	0.00064	0.00060	0.00063	0.00065	0.00064	3.34%	0.00059	8.51%
5	0.00214	0.00195	0.00204	0.00224	0.00200	0.00228	0.00224	0.00213	5.73%		
10	0.00477	0.00479	0.00483	0.00498	0.00472	0.00504	0.00505	0.00488	2.60%		

## Data Availability

The data that support the findings of this study are available upon request from the corresponding author.
